# Identifying scoliosis in a population-based adult cohort: automation of a validated method based on total body dual energy X-ray absorptiometry scans

**DOI:** 10.1007/s00586-025-09707-x

**Published:** 2026-01-28

**Authors:** Emmanuelle Bourigault, Amir Jamaludin, Timor Kadir, Andrew Zisserman, Jeremy Fairbank, Emma M. Clark

**Affiliations:** 1 Department of Engineering, University of Oxford, Oxford, United Kingdom; 2 Nuffield Department of Orthopaedics, Rheumatology and Musculoskeletal Sciences, University of Oxford, Oxford, United Kingdom; 3 Musculoskeletal Research Unit, Bristol Medical School, University of Bristol, Bristol, United Kingdom

**Keywords:** Deep Learning, Scoliosis, Geometry, Epidemiology

## Abstract

**Purpose:**

Scoliosis is a three-dimensional spinal deformity that, if left untreated, can progress to painful disability and require invasive surgical correction. In this work, we propose an algorithm to automate the established manual DXA Scoliosis Method (DSM) for measuring scoliosis in adults, that simultaneously quantifies **curve angle**, **direction** and **apex location**.

**Method:**

The automation pipeline comprises two stages: (i) segmentation of the spine and computation of its spine curve, and (ii) geometric analysis of that curve to identify the apex and derive the maximum modified-Ferguson angle as a continuous measure of severity. The automated method is validated for accuracy and repeatability. The validation is by a direct comparison with manual DSM measurements on 1,929 UK Biobank scans. The repeatability is from a test–retest analysis on a second DXA scanning session acquired 12 months after the primary scanning session for 2,728 participants.

**Results:**

Automated angles show excellent concordance with manual readings (Pearson r = 0.9; median bias = -1.8$^\circ$, 95% limits = $\pm 5^\circ$). Classification performance for key curve descriptors is high: location (specificity = 0.9), direction (0.8), and single- vs double-curve type (0.7). Validation between two sessions for the same patients reveals good agreement with a mean difference between sessions of 0.2$^\circ$.

**Conclusions:**

Given that DXA scanning is fast, inexpensive, and easily collected in large cohorts (e.g. UK Biobank and Avon Longitudinal Study of Parents and Children), the automated DSM enables population-scale spinal curvature phenotyping. This advancement will support robust scoliosis epidemiology, genetics, and natural history, potentially informing evidence-based screening strategies.

**Supplementary Information:**

The online version contains supplementary material available at 10.1007/s00586-025-09707-x.

## Introduction

Scoliosis is a spinal deformity that, if left untreated, can lead to long-term back pain and may eventually require invasive surgery [1, 2]. Though Adolescent Idiopathic Scoliosis (AIS) primarily affects adolescents [3], scoliosis in adults is not uncommon. It may develop later in life due to degenerative changes in the spine, such as disc degeneration, osteoarthritis and vertebral body collapse. Alternatively, the scoliosis found in adults may be prior AIS that was not surgically stabilised/corrected as it either was not large enough, surgery was declined or it was overlooked in adolescence.

The Cobb Method was introduced by Cobb [4] in his 1948 lecture series, and remains the gold standard for quantifying scoliosis curve severity. Despite its widespread adoption, studies report inter-observer variability of 3$^\circ$ to 10$^\circ$ and intra-observer variability of 2$^\circ$ to 7$^\circ$, limiting reproducibility [5, 6]. Efforts to standardize measurements include digital tools [7], yet manual assessments still dominate clinical practice. In addition, repeated radiographs pose long-term risks due to radiative exposure, necessitating low-dose alternatives. Dual-energy X-ray absorptiometry (DXA) offer a lower radiation exposure but may underestimate curves due to supine positioning [8–11]. A 6$^\circ$ threshold is recommended for DXA-based scoliosis diagnosis [8, 12], though debate persists about its sensitivity compared to standing radiographs.

Recent advancements in deep learning have significantly enhanced the automation of Cobb angle measurements from X-ray images, a critical factor in diagnosing and monitoring scoliosis. Several studies have explored various methodologies to improve the accuracy and reliability of these automated measurements [13–15].

The DXA Scoliosis Method (DSM) [9] is a validated method for measuring scoliosis from DXA scans. We explain the method with further details in subsection. The goal of this research is to fully automate the DSM measurement method for population-based epidemiological studies. This automation enables the angle, anatomical location, and direction of the spinal curve to be automatically determined from DXA scans in the UK Biobank adult cohort, providing standardized measurements suitable for large-scale research while acknowledging that clinical decision-making requires standing radiographs.

## Methods

### Study population

While most automated methods for scoliosis measurement have focused on AIS [16–18], this study measures scoliosis in adults using DXA scans from the UK Biobank [19]. UK Biobank is a large open-access medical dataset taken from over 500,000 volunteers aged 40 to 80 years. The data includes data related to imaging, genetics and health-related outcomes.

To develop the automation a selection of total body DXA scans were taken from the UK Biobank. In total, 48,384 DXA scans are available. An enriched sub-sample of 1,929 scans was identified from those available and who self-reported osteoarthritis to ensure a reasonable proportion of degenerative scoliosis.

In the UKBiobank, 2,416 volunteers were scanned at two time points less than a year apart. Because these sessions are so closely spaced, we expect minimal changes in the scoliosis curve/s as scoliosis in adults deteriorates slowly in general, regardless of its cause (degenerative or previous AIS) [1, 2].

### DXA imaging and positioning

DXA imaging is performed with patients supine, which eliminates gravitational effects on spinal curvature. Studies show supine measurements typically underestimate curve severity by 5–10$^\circ$ compared to standing radiographs [10, 11]. This limitation is acceptable for population-based epidemiological studies but precludes use for individual clinical decision-making.

### Manual annotation

All of 1,929 DXA scans were annotated, and 308 had scoliosis as defined by a maximum modified Ferguson angle of 6$^\circ$ or more. For curve location, we consider the full spine from C1-L5 containing 24 vertebrae. The Cervico-Thoracic ($\sim$C1-T11) and Thoraco-Lumbar ($\sim$T12-L4) categories are combined together such that Thoracic contains now Cervico-Thoracic and Thoracic categories (n=138) and Thoraco-Lumbar contains both Thoraco-Lumbar and Lumbar categories (n=175) (see Table 1).

**Table 1:** Distribution of Manual Annotations for Curve Patterns. Numbers (N) and percentages (%) out of the 308 that have scoliosis are shown in separate columns. Manual DSM annotated respectively the curve direction for right and left curves, the curve location for cervico-thoracic, thoracic, thoraco-lumbar and lumbar, and the curve type for C- and S-shape curves from DXA scans

Curve Type				Curve Location								Curve Direction			
C-shape		S-shape		Cervico-		Thoracic		Thoraco-		Lumbar		Right		Left	
				Thoracic				Lumbar							
N	%	N	%	N	%	N	%	N	%	N	%	N	%	N	%
286	92.9	22	7.1	1	0.3	132	42.9	41	13.3	134	43.5	171	55.5	137	44.5

### DXA Scoliosis Method (DSM)

**Fig. 1:**
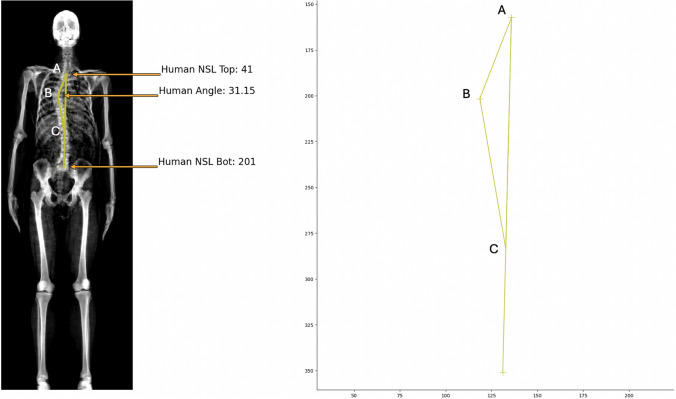
DXA Scoliosis Method (DSM). We show how the maximum angle of the spine is measured manually using the DSM. First the normal spine line (NSL) is drawn from the centre of the spine level with the first rib attachment to the centre of the spine at L5. Note that the graph presented was produced from the automated output. Then, the apex of the spine curve (**B**) corresponding to the maximum point of deviation from the NSL is marked. A triangle is formed by connecting the apex (vertex B) to vertices **A** and **C** respectively lying on the NSL. Vertices A and C are located where the center of the spinal column first touches the NSL on return from the apex. The spine curve angle is calculated as: 180$^\circ$ - $\hat{B}$

The previously published manual DSM [9] uses a modified-Ferguson method: a *normal spine line* (NSL) is drawn through the centre of the spine level with the first rib attachment, down to the centre of the spine at L5. Then, the apex of the curve is identified. Lines are drawn from the apex of the curve to the NSL at the point where the centre of the spinal column first touches the NSL on return from the apex. For double curves, the center of the spinal column at the judged point of inflection is used as the end of the curvature. The angle is calculated from these lines (see Fig. 1).

### Automated DSM

To automate the DSM we develop a three-stage approach that transforms DXA scans into clinically relevant measurements. First, we extract the spine curve from the DXA images using segmentation and curve fitting techniques to obtain a continuous representation of the spine midline. Second, we apply geometric principles to automatically compute the maximum angle from the extracted curve, mimicking the manual DSM procedure. Finally, we analyze the curve patterns to determine key clinical characteristics including curve direction, location, and type (C-shape vs S-shape).

#### Spine to curve: DXA spine segmentation and curve fitting

To extract the spine curve from DXA scans, we use previous published work [20, 21] that showed that the spine curve could reliably be obtained from DXA scans in the UK Biobank (implementation details available in Appendix 1).

#### Curve to angle: using geometry for angle prediction

**Fig. 2:**
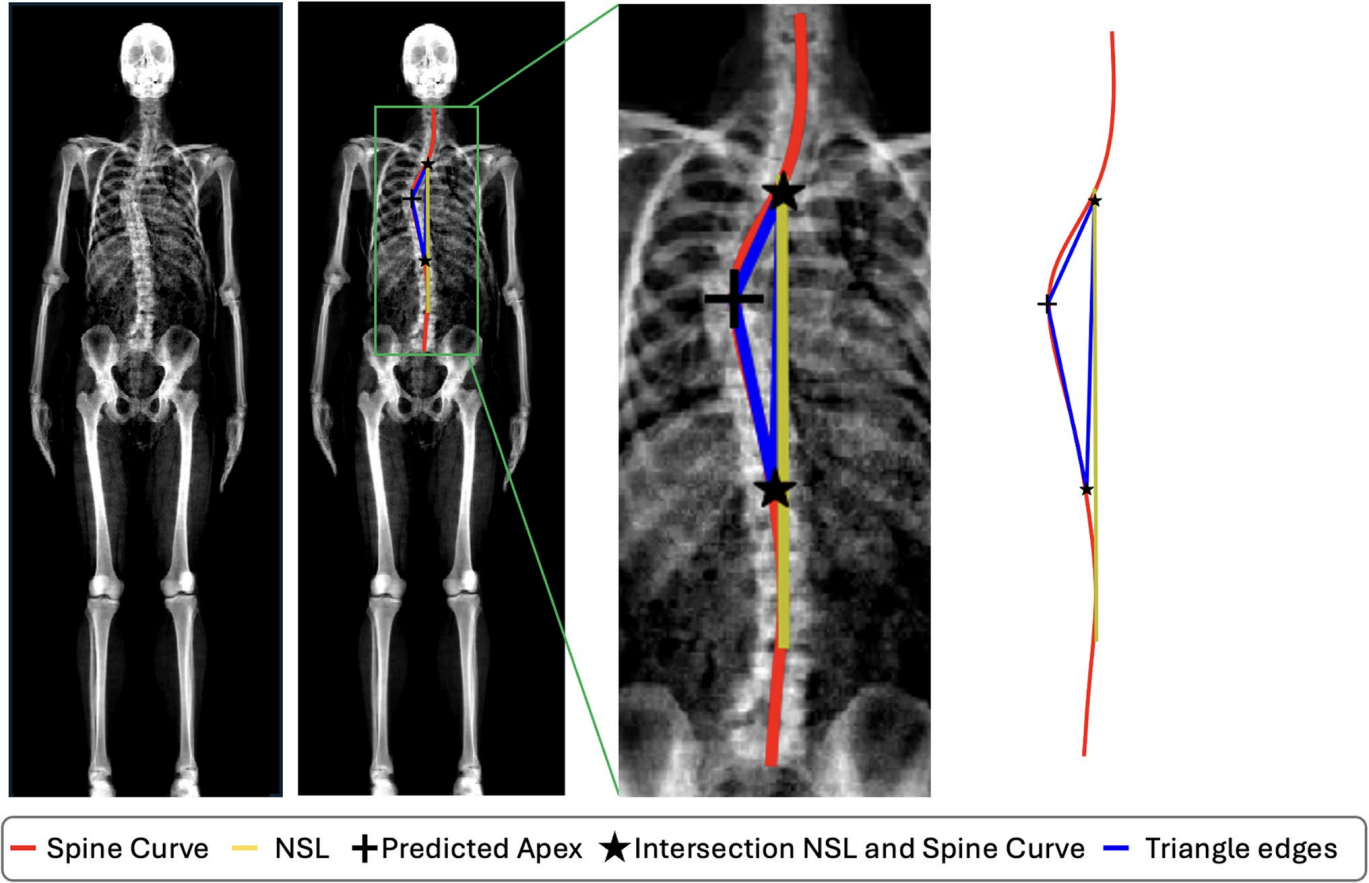
Automated Spine Curve Angle Computation. Our automated method reproduces the DSM method to measure the apex of the spine curve. We draw the lines (blue) going from the intersection (black stars) of the spine curve (red) and estimated normal spine line (yellow). The intersection of the blue lines is the curve apex (black cross)

Once the spine curve is defined, the manual procedure of measuring the spine curve angle was automated, as illustrated in Fig. 2. The procedure of calculating the spine curve angle by automating the manual DSM can be divided into two steps. First, we estimate the top/bottom coordinates of the NSL. This consists of taking the top and bottom of the spine segmentation by thresholding it to a binary segmentation map and finding the index of maximum and minimum points. Then, we calculate the spine curve angle using the triangle shown in Fig. 2.

#### Curve patterns

##### Curve direction

**Fig. 3:**
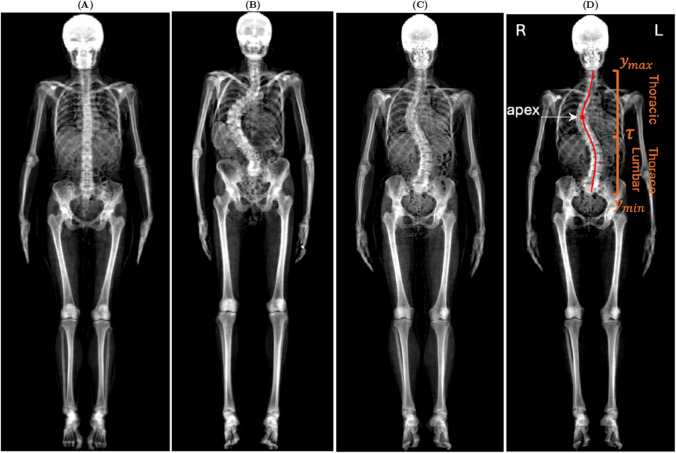
Curve pattern explanatory figure. We show in (**A**) a straight spine, in (**B**) a C-shape spine curve, (**C**) an S-shape spine curve, and (**D**) an S-shape spine curve for direction and location explanation. The apex of the largest spine curve is selected as the primary apex and is indicated by a white arrow. We indicate the patient’s right (R) and left (L) side. The direction is determined by the location of the primary apex pointing to the right or left of the patient. This corresponds to the sign of the curvature of the curve. We present curve location in terms of threshold $\tau$ to distinguish the thoracic and thoraco-lumbar regions using spine height values in [$y_{min}$,$y_{max}$]

From the spine curve extracted in "Spine to curve: DXA spine segmentation and curve fitting", we can automatically predict *right* curve direction corresponds to the right side of the participant (left side of DXA scan) while *left* curve direction corresponds to the left side of the participant (right side of the DXA scan) as shown in Fig. 3.

##### Curve location

The location of the curve can be determined using the y index of the spine curve. In our settings, our model aims to predict the thoraco-lumbar region which encompasses the bordering region between thoracic and lumbar and the lumbar region (see Fig. 3). We measure the location of the largest spine curve’s apex by estimating a dynamic threshold value $\tau$ on the vertical axis optimised to separate the thoracic from the thoraco-lumbar region. We opted for binary classification since few of the annotated sets are effectively in the thoraco-lumbar region and this region is challenging to delimit by humans (see Table 1).

##### Curve type 

The spine curves vary in type depending on the number of extrema. A spine curve with one apex is classified as C-shape curve or equivalently single curve while a spine curve with two apexes is classified as S-shape curve or double curve. Manual annotators have annotated the curves with the number of apexes. The challenge is to correctly identify the S-shape curves since the angle values per scan comprise several local extrema. S-shape by definition has two apexes differing in signs.

According to the manually annotated set of 308 scans, 286 scans have been annotated as having one curve or C-shape, and 22 as double curves or S-shape. To ensure proper training, the dataset was stratified into training, validation, and test sets using an 80-10-10 split. The training set contains 246 entries (with 228 annotated as C-shape and 18 as S-shape), while the validation set includes 31 entries (29 C-shape and 2 S-shape), and the test set comprises 31 entries (29 C-shape and 2 S-shape).

Let’s denote as $\theta _{1}$ the primary angle of the spine curve and $\theta _{2}$ the secondary angle of the spine curve. For analysis of the correspondences in values, we make sure all $\theta _{1}$ are positive and $\theta _{2}$ are negative. The challenge is to correctly identify the S-shape curves since the angle values per scan comprise several local extrema. S-shape by definition has two apexes differing in signs. How big in value do the two apexes have to be to avoid local extrema while capturing the relevant apexes? This is the investigation we show in results curve patterns in "Curve patterns (location, direction, type)".

In order to determine curve type automatically, we follow the following steps. First we compute the primary apex and secondary apex of the spine curve by finding the point of biggest deviation of the spine curve relative to the NSL (see Fig. 2). Note that the secondary apex is chosen pointing in the opposite direction to the primary apex. Then, we measure how big in value the two apexes are. Based on manual annotations, we define a threshold for the minimum value of the two apexes to classify S shape curves from C shape curves.

### Evaluation metrics

The association between automated DSM angles and manual DSM angles is measured with Bland-Altman analysis: for each paired measurement we plotted the difference (automated – manual) against the mean of the pair.

To quantify performance between the automated DSM method and the ground-truth annotation for curve pattern predictions, we compiled a $2\times 2$ confusion matrix that tallied true-positives (TP), false-positives (FP), false-negatives (FN) and true-negatives (TN). From these four cells we derived: *sensitivity*, the proportion of actual positives correctly identified; *specificity*, the proportion of actual negatives correctly identified; positive predictive value *PPV*, the probability that a positive test truly represents either the direction i.e right or left or location i.e thoracic or thoraco-lumbar of the apex; and negative predictive value *NPV*, the probability that a negative test truly represents absence of that direction or location of the apex.

To deal with our imbalanced dataset in class distribution, we employ the balanced accuracy which calculates the average of sensitivity and specificity.

For the curve categorisation prediction, we employ the AUC (Area Under the Curve), which is used for evaluating the performance of our binary classification model for single (C-shape) or double curve (S-shape) spines. The AUC value ranges from 0% to 100%, with higher values indicating better model performance.

## Results

### Automated spine curve angle

**Fig. 4:**
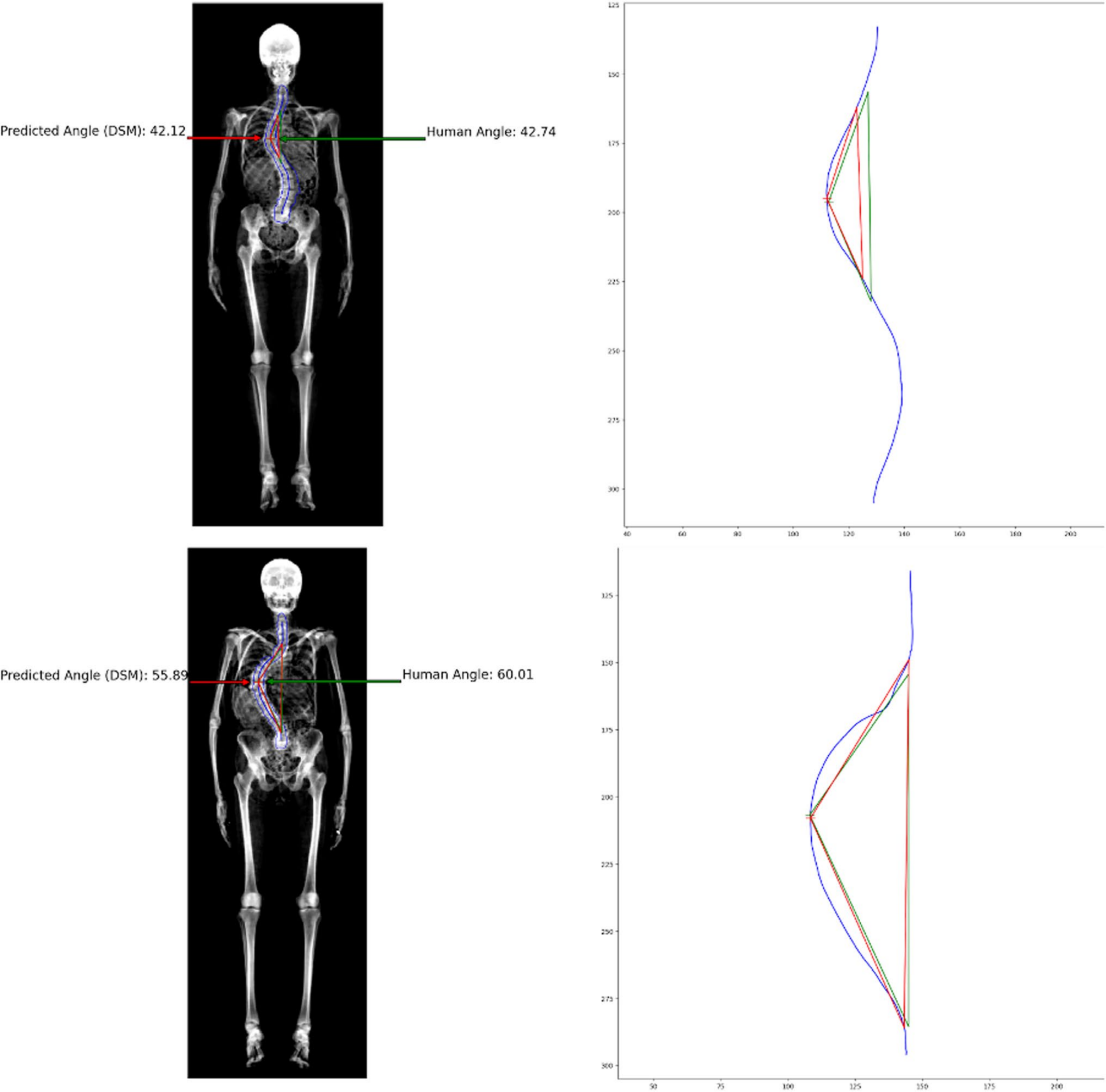
DSM automated angles. An example of our automated DSM angles from estimated triangles with red for automated and green for manual DSM ground-truth. First, the spine is segmented (blue contour) and the apex of the curve computed using the pipeline in Appendix 1.1. The top and bottom vertices of the triangle are estimated relative to the height of the patients, see explanatory diagram in Fig. 2

**Fig. 5:**
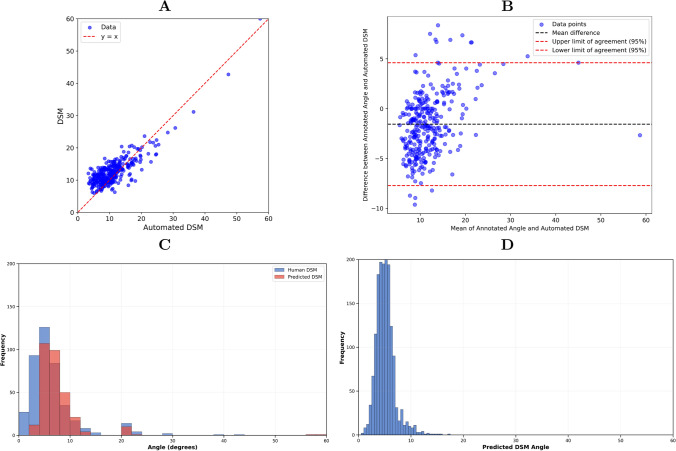
Spine curve angle agreement between manual and automated methods. **A** Compares manual DSM with automated DSM spine curve angles ($\rho$ = 0.9, n = 308). **B** Shows Bland-Altman of automated versus manual DSM spine curve angles (degree) with mean difference of −1.6 and median difference of −1.8 and 95% CI of [−7.7, 4.6]. **C** Histogram showing the distribution of automated DSM angles with our method on the annotated and manual DSM angles on the set of scoliosis cases (n=308). **D** Histogram showing the distribution of automated DSM angles with our method on the annotated set of normals (n = 1,621; not annotated for DSM angle i.e. modified Ferguson angle)

Our automated spine curve angle values are strongly associated with manual DSM values with Pearson’s R of 0.9 (see Fig. 5). The mean difference between the automated and the manual DSM spine curve angle values is in the range [−7.7, 4.6] in degrees with 95% confidence (see Fig. 5). We observed from visual inspection of the automated triangles (red) versus manual DSM triangles (green) that our automated triangles are on average smaller than manual (see Figs. 4 and 5).

Our automated spine curve angle values are strongly associated with manual DSM values with Pearson’s R of 0.9 (see Fig. 5). The mean difference between the automated and the manual DSM spine curve angle values is in the range [−7.7, 4.6] in degrees with 95% confidence (see Fig. 5). We observed from visual inspection of the automated triangles (red) versus manual DSM triangles (green) that our automated triangles are on average smaller than manual (see Figs. 4 and 5).

To take into account the differences in the procedures between the manual and automated DSM, we consider a set of lateral shifts and take the median of automated spine curve angles as the final automated spine curve angle. The resulting correlation and mean difference are available in Fig. 5 panels C and D. The DSM method (manual) has previously been shown to be reliable where 95% of repeat measures were within 5$^\circ$. This suggests that our automated DSM preserves the error range of manual curve angle measurements (5$^\circ$) and therefore suggests that the automated curve angle measurements show good agreement with the manual measurements.

#### Verification of straight spines

To validate our automated DSM, we apply it to the set of straight spines n = 1,621 ($<6^{\circ }$) from the set annotated by manual reviewers. We report the distribution of automated DSM angles with our method scans in the histogram (see Fig. 5D). The number of scans DSM angles above 6 degree from our automated method is n = 431 (26.6%). On average, we have n = 61 (3.8%) above the scoliosis threshold using the underprediction of −2.9 degrees with manual annotation.

### Curve patterns (location, direction, type)

#### Curve location

**Fig. 6:**
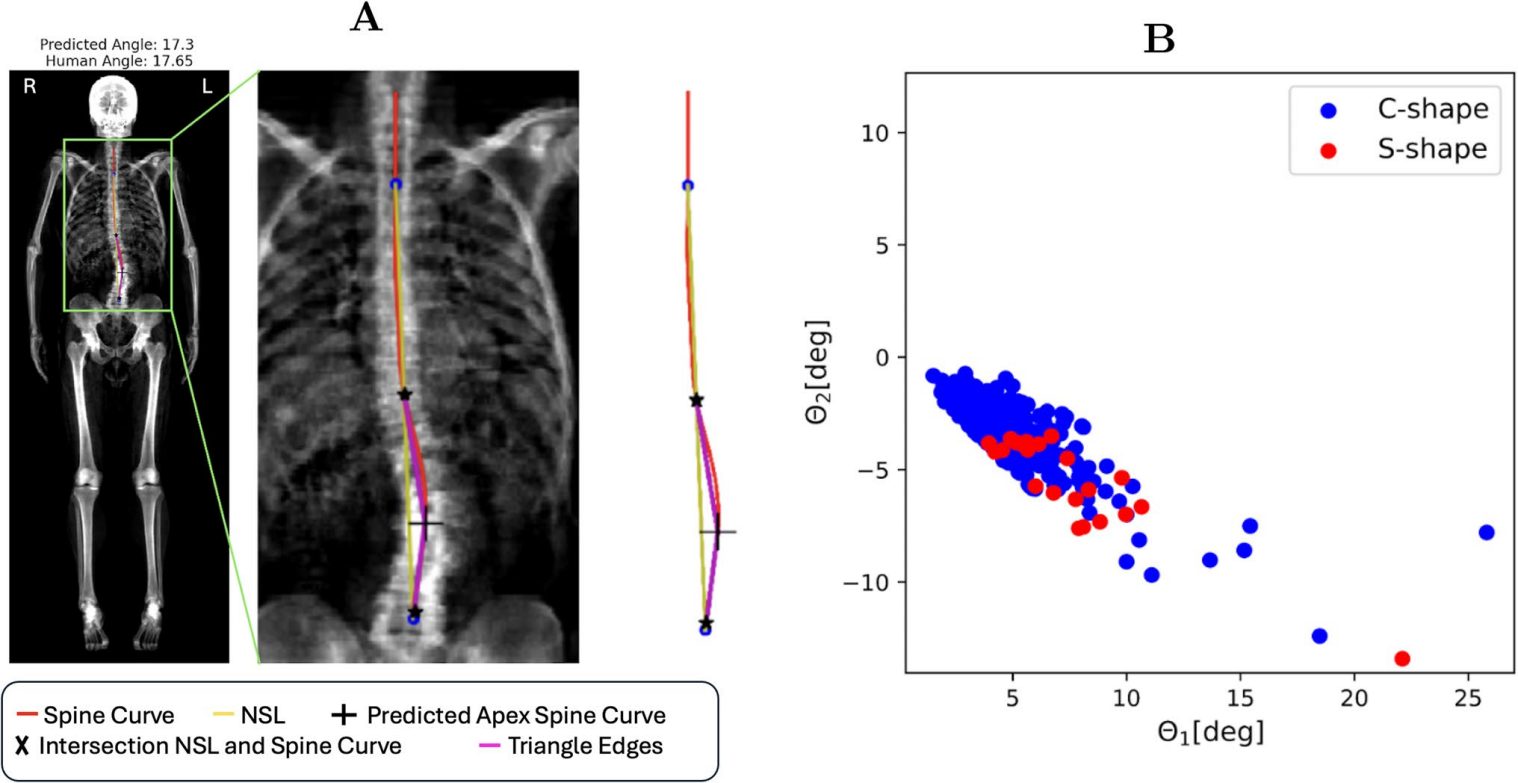
Curve direction prediction. **A** Diagram showing the apex of the curve (black) and triangle (magenta) within the apex region (green). **B** Scatter plot of the primary angle of the spine curve $\theta _{1}$ versus the secondary angle $\theta _{2}$. All $\theta _{1}$ are made positive, while all $\theta _{2}$ are made negative for simplicity as we are interested in the magnitude of angle values

 For curve location prediction, our automated model achieves a balanced accuracy of 0.8, specificity of 0.9, sensitivity 0.6, PPV 0.9 and NPV 0.6 (see Fig. 6A and Table 2).

#### Curve direction 

For the curve direction prediction, our automated model achieves a balanced accuracy of 0.7, Specificity of 0.8, Sensitivity of 0.7, Predictive Positive Value (PPV) of 0.8 and Negative Predictive Value (NPV) of 0.7 (see Fig. 6A and Table 2).

**Table 2:** Confusion Matrices for Spine Classification. Note that thoraco-lumbar class encompasses the original two categories, thoracic and thoraco-lumbar curves

Thoracic vs Thoraco-lumbar			Left vs Right		
True labels	Predicted labels		True labels	Predicted labels	
	Thoracic	Thoraco-lumbar		Left	Right
Thoracic	113	15	Left	109	31
Thoraco-lumbar	74	105	Right	54	114

We show the predictions of the model for right/left against labels on the set of 308 annotated for manual DSM angle. We repeated the evaluation with the cases of angles close in value. We checked whether the secondary apex is in the apex region (green box region) defined by inflection points on the spine curve (black stars) and closer in distance to the manual DSM apex. We obtain a balanced accuracy of 0.9. We explored including in the evaluation the secondary apex, the accuracy of our method jumped from 72.4% to 92% suggesting that a significant cause for the reduced performance of the automated DSM was the cases with S shaped spines where both curve angles were of a similarly small magnitude. There are ambiguous cases where our method failed to predict the correct direction of the spine curve. These are mainly cases with detected double curves with two apexes close in values. We observed that failures mainly occur for smaller curve angles, where the two main apexes (absolute value) are close in value ($< 2^{\circ }$ difference). These cases are close in angle values but differ in the pointing direction of the apex.

#### Curve type

We employ a classifier to distinguish between C-shape and S-shape as described in "Curve location".

From Fig. 6 B, the ratio $\theta _{1}$ to $\theta _{2}$ is bigger in magnitude on average for S-shape curves (red dots) than for C-shape curves (blue dots). However, there is a region of overlap of the red and blue dots suggesting there is no clear determination of the number of apexes in the curve by considering the primary and secondary apex in the spine curve only. To address the class imbalance, we weigh the classes in the loss function. The performance of our curve type prediction model is evaluated on the test set, where we achieve an AUC of 61.1%.

### Validation of spine to spline and Curve patterns on two UKBB sessions

We validated our automated DSM angles for two sessions taken less than a year apart for the same participants. In our evaluation, we observe a strong association between the two sessions, with a mean difference of just 0.2$^\circ$ between Session 2 and Session 1 (see Fig. 8 for the Bland-Altman analysis, which reports a 95% confidence interval around the mean difference of [−3.9, 4.3]). Paired samples t-test is 2.5, p-value: 0.012 which shows no significant difference between sessions (p $> 0.05$). The effect size (Cohen’s d for paired data) is 0.06 which we can interpret as being negligible.

Furthermore, we assess the correspondence in curve patterns as additional validation. For both sessions, the percentage distribution of curve directions is nearly identical: in Session 1, 58.1% of curves were left and 41.9% were right, compared to 58.1% left and 41.9% right in Session 2.

**Fig. 7:**
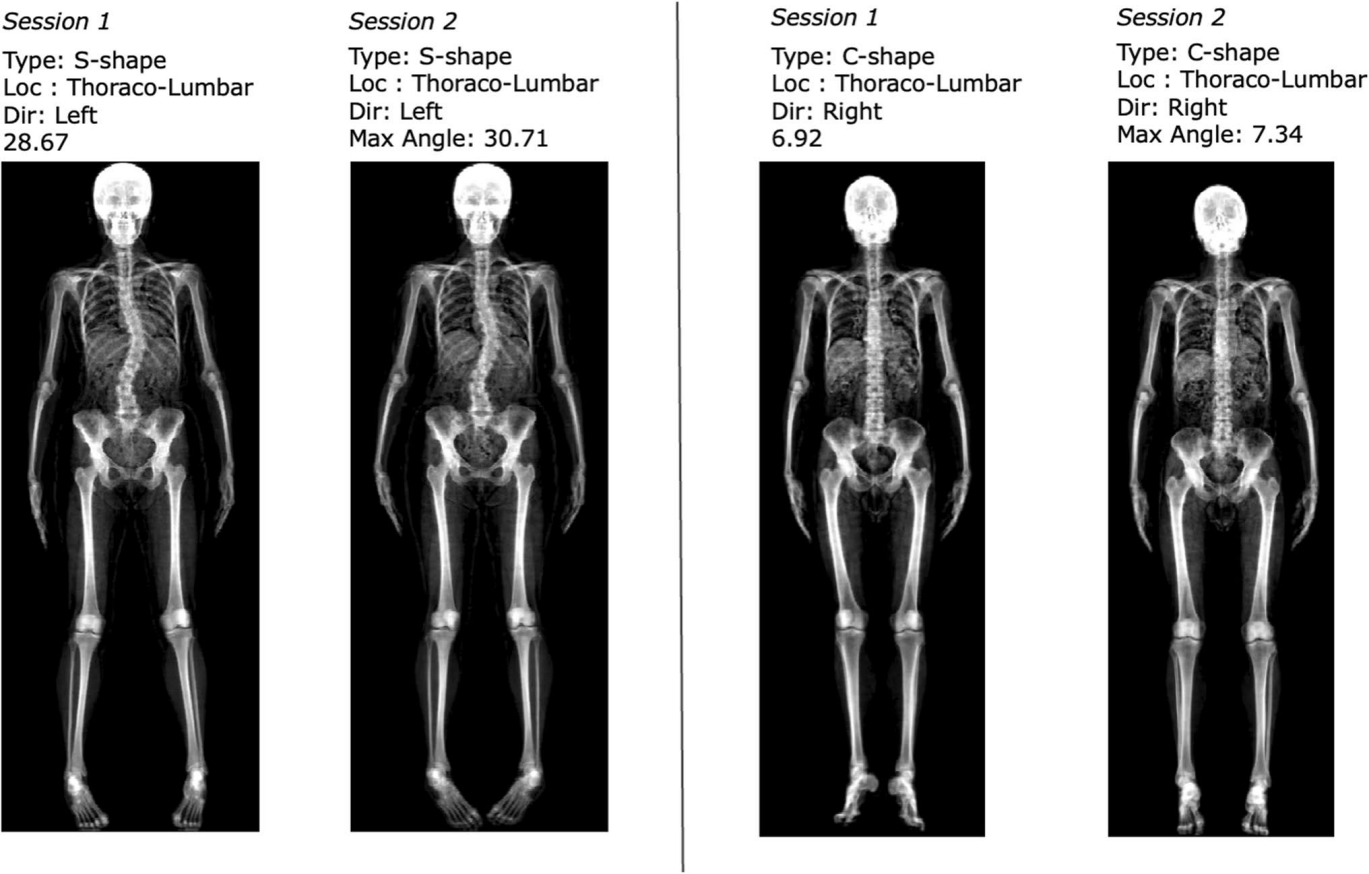
Curve Pattern Validation for Two Sessions. We show 2 examples for curve pattern predictions i.e. type, location, direction, and maximum angle for session 1 (*left*) and session 2 (*right*) DXA scan taken a year apart

**Fig. 8:**
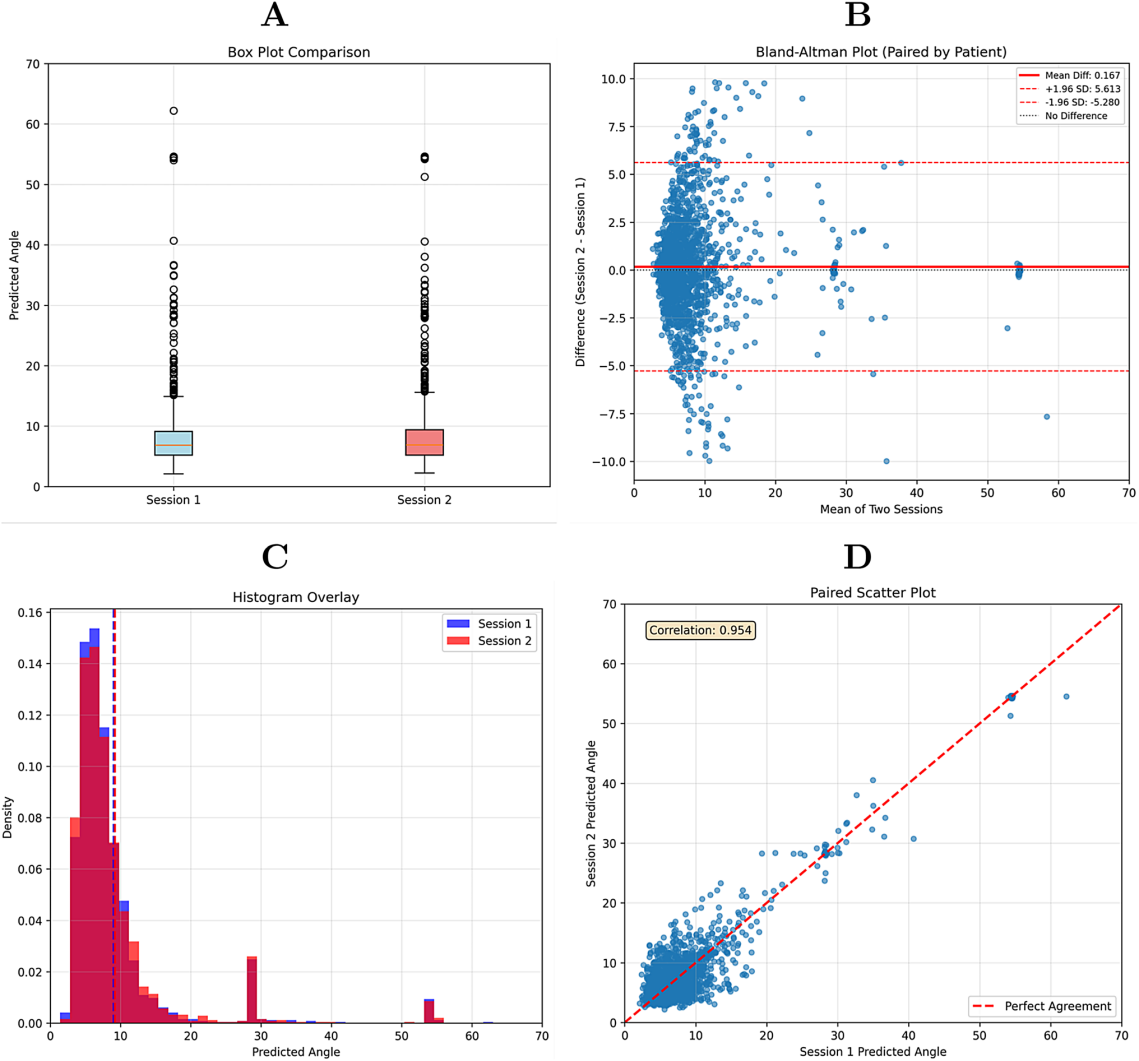
Comparison of DSM automated angles in degrees between two sessions (n=2,416). We show the distribution of automated DSM angles for the two sessions in different forms, starting from panel A with boxplots, panel B Bland-Altman between two Sessions where the mean difference is 0.2 and 95% confidence interval around the mean difference is [−3.9,4.3]. Panel C shows the histograms of the two session and panel D the scatter plot with correlation of 0.96 in automated DSM angles between the two sessions

Similarly, the location of the apex is preserved across sessions. In session 1, 39.6% of the apexes were thoracic and 60.4% thoraco-lumbar, which is comparable to the 39.5% thoracic and 60.5% thoraco-lumbar distribution observed in Session 2. Although the size of the curves might evolve slightly over this short period, the number of apexes is very stable. Both sessions report 92.3% C-shaped curves and 7.7% S-shaped curves (see example of predictions for two sessions in Figs. 7, 8).

## Discussion

This study presents an automated geometric method for measuring scoliosis parameters from DXA scans in a large adult cohort, achieving strong concordance with manual measurements (Pearson r = 0.9, mean bias = $-1.6^\circ$). The automated DSM demonstrated excellent test-retest reliability across sessions with a mean difference of $0.2^\circ$ and high classification performance for curve location (specificity = 0.9) and direction (accuracy = 0.8). These findings suggest that automated DXA-based assessment can provide reliable population-level scoliosis phenotyping, though several important limitations must be carefully considered.

The performance of our automated method compares favorably with recent advances in deep learning for scoliosis measurement. [16] reported mean absolute errors of $3.8^\circ$ for automated Cobb measurements from standing radiographs, while [17] achieved $2.9^\circ$ using vertebral landmark detection. Our mean difference of $-1.6^\circ$ with 95% limits of agreement of ±5$\circ$ is comparable to these standing radiograph-based methods, despite the inherent challenges of supine imaging. This level of accuracy falls within the clinically acceptable range of inter-observer variability of $3-10^\circ$ reported by [5] and the intra-observer variability of $2-7^\circ$ documented by [6]. Notably, our approach differs by employing the modified-Ferguson method rather than Cobb angles, prioritizing reproducibility for epidemiological applications over direct clinical measurement.

A limitation of our approach, is the supine positioning inherent to DXA imaging. For epidemiological purposes, this limitation may be acceptable given the standardization it provides. [22] demonstrated that while absolute measurements differ between supine and standing positions, curve progression patterns remain detectable in serial supine imaging, suggesting utility for longitudinal population studies. The validation by [9] of a $6^\circ$ threshold for DXA-based diagnosis specifically accounts for supine underestimation, and our findings of consistent curve patterns between sessions support the method’s reliability for population research rather than individual clinical decision-making.

The choice of the modified-Ferguson method over the traditional Cobb angle measurement, while departing from clinical convention, offers specific advantages for automated analysis. Ferguson [23] originally developed this technique for cases where vertebral endplates were difficult to visualize, a challenge particularly relevant to DXA imaging where vertebral detail may be limited. Stokes [24] demonstrated that Ferguson angles correlate strongly with Cobb angles (r = 0.95) while showing superior inter-observer reliability in automated systems. This improved reproducibility is crucial for large-scale epidemiological studies where standardization across thousands of measurements takes precedence over absolute clinical accuracy for individual patients.

Regarding radiation exposure, the advantages of DXA for population screening become apparent when considering dose comparisons. [25] reported whole-body DXA effective doses of 4.6 $\mu$Sv, compared to 700–1000 $\mu$Sv for standard posteroanterior spine radiographs. This 150-fold reduction in radiation exposure makes DXA particularly suitable for repeated measurements in longitudinal studies, addressing the ALARA (As Low As Reasonably Achievable) principle advocated by [26] for medical imaging.

The reduced performance in detecting S-shaped curves, with an AUC of only 61.1%, represents a current limitation of our work. This challenge arises from multiple converging factors. The severe class imbalance in our training dataset, with only 7.1% of cases presenting S-shaped curves, reflects the natural distribution in adult populations where [27] found double curve prevalence of 5–10%. This imbalance poses well-documented challenges for machine learning algorithms, as comprehensively reviewed by [28] in their analysis of learning from imbalanced data.

The age distribution of our cohort further compounds the S-curve detection challenge. Unlike adolescent idiopathic scoliosis where double major curves are more common, as documented by [29], adult degenerative scoliosis typically presents as single lumbar or thoracolumbar curves, as characterized by [30]. Our adult cohort from the UK Biobank, naturally exhibits fewer S-shaped presentations, making algorithm training more challenging. Future iterations could benefit from synthetic minority oversampling techniques, as demonstrated by [31] for rare scoliosis pattern detection, potentially improving classification performance for these less common curve types.

The integration of our automated method with existing UK Biobank infrastructure represents a significant methodological advancement. Building upon the work of [20] who demonstrated automated quantification of spinal curve size from DXA scans, our method extends the utility of these population-scale imaging resources to comprehensive scoliosis assessment. This approach aligns with the principles established by [32] in natural history studies, which demonstrated that population-based scoliosis research requires large cohorts with standardized measurements rather than clinical precision for individual patients.

Our method offers unique advantages for population-based research. The complete automation eliminates inter-observer variability, a persistent challenge in manual Cobb angle measurement that has limited the reproducibility of epidemiological studies. The scalability of the approach enables analysis of tens of thousands of scans, far exceeding manual capacity and opening new possibilities for genetic association studies, as suggested by [33] in their analysis of scoliosis heritability. Furthermore, the standardization achieved through automation facilitates multi-center studies and long-term longitudinal analyses essential for understanding disease natural history and identifying risk factors for progression.

Looking forward, several avenues could enhance the clinical utility of DXA-based scoliosis assessment. Development of statistical models to estimate standing angles from supine measurements, building on the work of [34], could bridge the gap between population screening and clinical application. Incorporation of lateral DXA views, when available, could enable assessment of sagittal alignment and rotational components, as demonstrated by [35]. Advanced machine learning techniques, particularly attention mechanisms as implemented by [18], could improve detection of subtle curve patterns and enhance S-curve identification. Development of risk stratification algorithms could identify individuals requiring standing radiographic follow-up, creating a practical screening pathway from population-level assessment to targeted clinical evaluation.

## Conclusion

This automated DSM method successfully enables large-scale scoliosis phenotyping from existing DXA imaging resources, addressing a critical need for standardized measurement in population-based research. While supine positioning and the modified-Ferguson approach preclude direct clinical application for individual patient management, particularly for surgical planning in adolescent idiopathic scoliosis, the method serves its intended purpose for epidemiological investigation.

The method provides comprehensive quantitative assessment including curve location, direction, and extent, enabling rapid analysis of large datasets. It overcomes traditional limitations of X-ray-based manual measurement by offering an efficient, and low-radiation alternative suitable for population-scale screening.

Measurement discrepancies arise from methodological differences where the automated system uses extracted spine curves while human annotators estimate vertebral centers, creating x-axis deviations. Human annotation errors, particularly in challenging cases like double or smaller curves, contribute to variability. Some ambiguous cases required clinical revision, highlighting the complexity of visual assessment even for experts.

Using DXA scans to identify the presence of deformity has been shown to be successful and can be a reliable tool for screening. However, quantifying deformity in the supine position differs from standing measurements. Therefore, after using this method to screen patients for deformity, standing radiographs are recommended for accurate Cobb angle measurement when clinical decision-making is required.

The automated DSM pipeline that we have validated here is a step towards the feasibility of automated assessment for large-scale epidemiological studies.

## Supplementary Information

Below is the link to the electronic supplementary material.

Supplementary file 1 (pdf 3521 KB)

## Data Availability

Under UK Biobank agreement number 17295, all authors have access to the DXA scans (field ID 20158) used in this study. This dataset can be downloaded after creating an account and registering on the UKBiobank platform https://www.ukbiobank.ac.uk/use-our-data/apply-for-access/. The inference code and weights will be made publicly available via Github. Training code will be made available through the Biobank platform.
