# Supplementary material for: Identifying scoliosis in a population-based adult cohort: automation of a validated method based on total body dual energy X-ray absorptiometry scans
Source: Eur Spine J. 2026 Jan 28;35(7):3893–905. doi: 10.1007/s00586-025-09707-x (PMC13372959; doi:10.1007/s00586-025-09707-x)
Supplement: Supplementary file 1 — (pdf 3521 KB) [file 586_2025_9707_MOESM1_ESM.pdf]

Supplementary Material: Identifying Degenerative Scoliosis in a Population-Based Adult Cohort: Automation of a Validated Method Based on Total Body Dual Energy X-ray Absorptiometry Scans

1 Model Implementation Details

1.1 Spine Curve Extraction

A segmentation network and geometric approach is used to obtain the spine curve prediction of the spine given a DXA scan (see Figure 1 and details in [1]). All scans are height normalised and resized to consistent resolution (832 x 320).

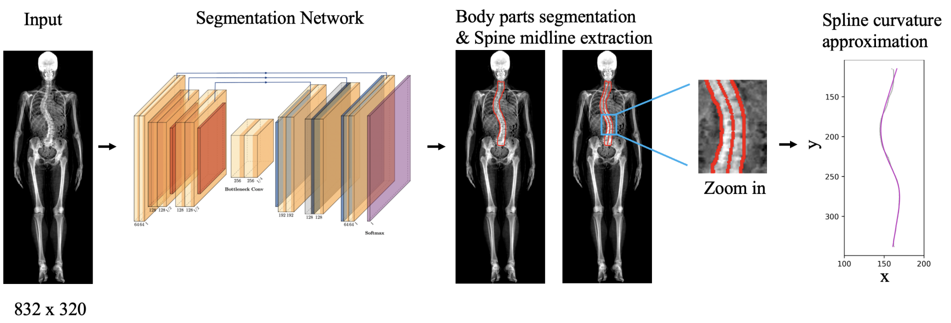

Fig. 1: **Pipeline of the automated scoliosis measurement method.** The segmentation network receives DXA scan (832×320) and ground-truth masks for 6 body parts: head, spine, pelvis, cavity, left leg and right leg. At each row of the spine probability map, the weighted arithmetic mean of the probability and the indices of the scores is calculated to be the predicted midpoint. Cubic spline approximation is finally employed to filter out noisy predictions.

The segmentation contours (Figure 2) were obtained using the U-Net model in Table 1 from [1]. The segmentation model follows the standard U-Net from [2]. The input is height normalised DXA scan (832,320) and targets are body masks for head, spine, cavity, right leg and left leg. The output of the segmentation model is (832,320,5)

where each of the 5 channels corresponds to mask output for the body parts. See Table 1 for details of the resolutions and channels, Figure 1 and Figure 2 for qualitative visualisations.

To obtain the spine curve: each midpoint along the spine is computed by taking the weighted average of pixel indices, where the weights are the softmax probabilities calculated from the model's confidence scores at each position. Cubic spline is fitted through the midpoints extracted from the mask of the spine.

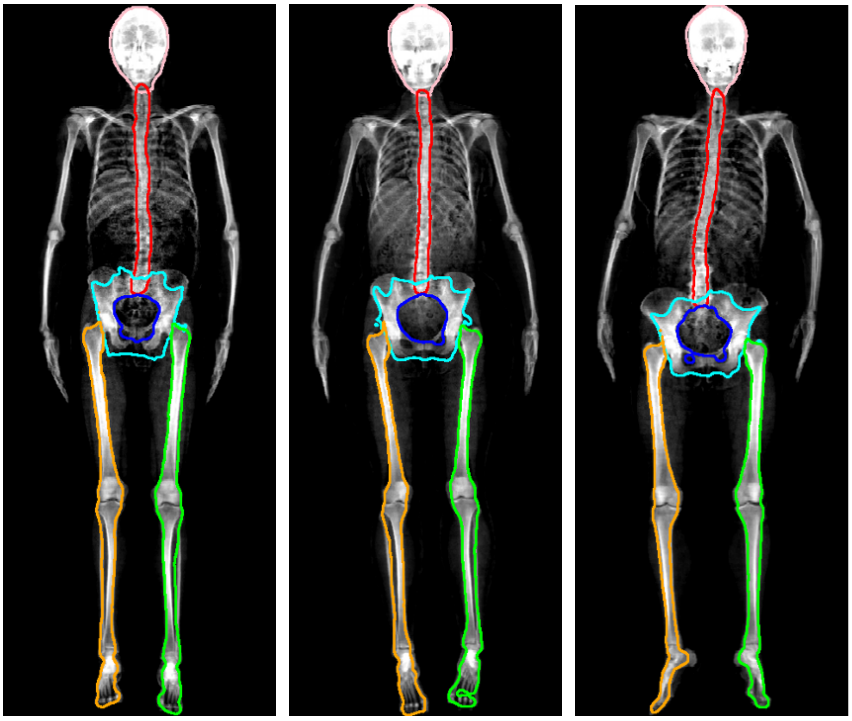

Fig. 2: **Body part segmentation for head, spine, cavity, pelvis, left leg and right leg.** These segmentation contours were obtained using the U-Net model in Table 1 from [1].

1.2 Automatic Positioning Error Estimation

According to the definition of positioning error by [3], using the automatic DSM method for primary manual DSM angle measurement, features such as pelvic obliquity or raised shoulder were classified as definite positioning error. Further visual inspection of scans were performed where curves were not clearly explained by poor body positioning. Then, in the next stage, scans were further classified as possible positioning error when it was impossible to classify the curve as either likely scoliosis or definite

Layer	Resolution	Channels
Input	832 × 320	1 (Grayscale)
Convolution Block 1	832 × 320	64
Bilinear Interpolation 1	416 × 160	64
Convolution Block 2	416 × 160	128
Bilinear Interpolation 2	208 × 80	128
Convolution Block 3	208 × 80	256
Bilinear Interpolation 3	104 × 40	256
Convolution Block 4	104 × 40	512
Bilinear Interpolation 4	52 × 20	512
Bottleneck	52 × 20	1024
Up Convolution 1	104 × 40	512
Convolution Block 5	104 × 40	512
Up Convolution 2	208 × 80	256
Convolution Block 6	208 × 80	256
Up Convolution 3	416 × 160	128
Convolution Block 7	416 × 160	128
Up Convolution 4	832 × 320	64
Convolution Block 8	832 × 320	64
Output	832 × 320	5 (Output Channels)

Table 1: U-Net architecture with layer-wise resolution and number of channels.

positioning error. Such cases included curve features that could explain scoliosis but some clearly visible body-positioning errors were identified.

During scan acquisition, the patient may not be lying still and consequently bending on one side. This lateral bending is hard to control in practice and can affect the measurement of angle and curvature. Examples of positioning error are shown in Figure 3. In the 2K annotation set, 1,693 DXA scans have been labelled as having clear positioning error. In the UKBiobank, a large proportion of scans have positioning error (87.7%) according to the sample of 1,929 DXA scans annotated by humans. The positioning error typically leads to a cervico-thoracic curve. In most cases, the shoulders or head are not aligned and scans with positioning error can be detected by measuring the angle of these body parts.

To automatically assess body positioning error in the cohort of the UK Biobank, a geometric method is proposed measuring angles for head alignment, shoulders alignment, spine endpoints alignment, pelvis alignment and leg symmetry (see Figure 3). A classifier with a single linear layer was trained on the set of 1,929 annotated scans for positioning error.

The five main input features given to the classifier are the following: head angle, shoulder angle, spine angle, legs symmetry, and pelvis angle.

- **Head Angle**: computed by finding the angle between the line of best fit through the head midpoints and the vertical line
- **Shoulder Angle**: computed by finding the angle between the line through the first non-zero shoulder row and the horizontal line

- **Spine Angle**: computed from the line joining the endpoints of the spine and the vertical line
- **Legs Symmetry**: computed from the lines through knees midpoints after horizontally flipping one knee to measure symmetry
- **Pelvis Angle**: computed by finding the angle between the horizontal line and the top and of the pelvis. The pelvis is segmented using pre-trained SAM by setting a bounding box around the pelvis region and fixing negative seeds (5 or less) manually to guide the segmenter [4]. The top line of the pelvis is obtained by joining two points, top y index of the left and right part of the pelvis mask.

The model is expressed by:

$$Z = W^T X + b$$

where Z defines a logistic-regression classifier, X corresponds to the input feature vector fed to the neural network, and b is the bias term. For each input feature X, there is a corresponding weight W, which signifies how strongly the effect of the input is on the output. Therefore, patients with positioning error above a certain threshold computed from output of the classifier are discarded after visual inspection of the scans following the DSM method (see Figure 3).

The softmax outputs are used to rank the scans from lower to higher scores. Then, expert clinicians visualised the scans with hierarchical ranking to select the optimal threshold with uncertainty.

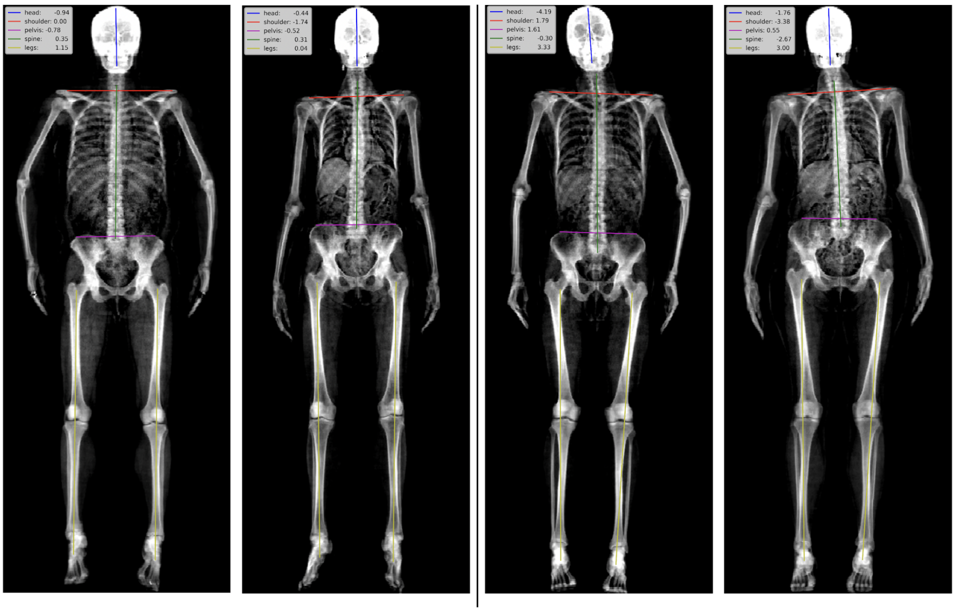

Fig. 3: **Visualisation of body angles and body positioning error.** Two cases of low positioning error < 0.25 (left) and high positioning error > 0.9 (right) with corresponding angles for head, legs, shoulders, spine and pelvis on top of the scan with lines used to draw the angle.

2 Experimental Analysis on Automated Angle Measurement

2.1 Scoliosis Severity Agreement

The angles obtained from the automated DSM given the segmented spine show good agreement with human annotation for spine curves varying in severity (see Figure 4).

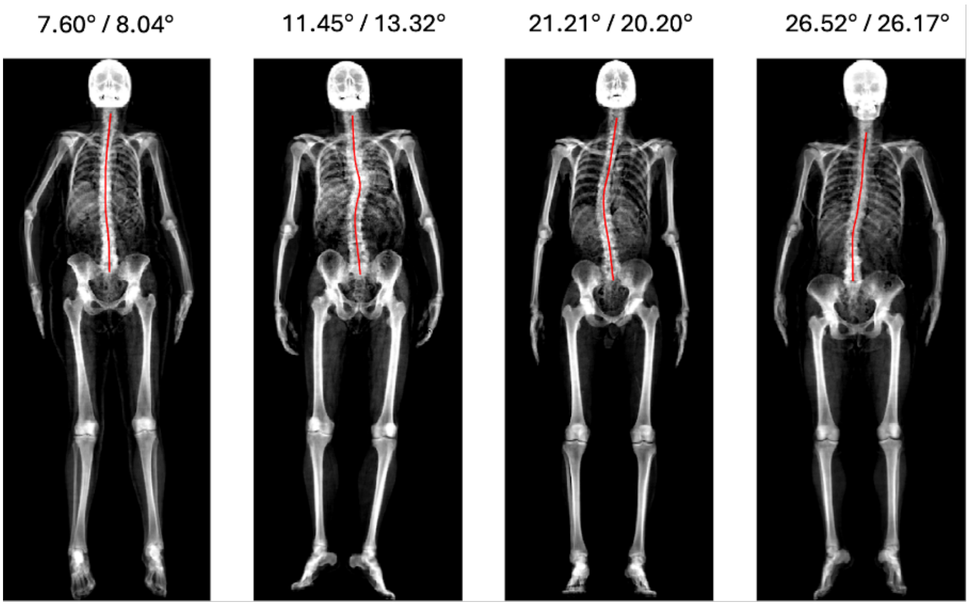

Fig. 4: **Automated DSM Visualisation of Scoliosis Severity.** Splines of the spine (red) overlayed on DXA scans for angles ranging from mild (left) to severe (right). On top of each scan: (i) automated DSM angle (degree), and (ii) human maximum angle annotated by expert clinicians.

References

[1] Bourigault, E., Jamaludin, A., Kadir, T., Zisserman, A.: Scoliosis measurement on DXA scans using a combined deep learning and spinal geometry approach. In: Medical Imaging with Deep Learning (2022)

[2] Ronneberger, O., Fischer, P., Brox, T.: U-net: Convolutional networks for biomedical image segmentation. In: MICCAI, pp. 234–241. Springer, ??? (2015)

[3] Taylor, H., Harding, I., Hutchinson, J., Nelson, I., Blom, A., Tobias, J., Clark, E.: Identifying scoliosis in population-based cohorts: Development and validation

of a novel method based on total-body dual-energy x-ray absorptiometric scans. Calcified Tissue International **92**, 539–547 (2013)

[4] Kirillov, A., Mintun, E., Ravi, N., Mao, H., Rolland, C., Gustafson, L., Xiao, T., Whitehead, S., Berg, A.C., Lo, W.-Y., *et al.*: Segment anything. In: Proceedings of the IEEE/CVF International Conference on Computer Vision (ICCV) (2023)
